# Mental Health as Assessed by the Symptom Checklist 90 (SCL-90) Scores in Women with and Without Polycystic Ovary Syndrome

**DOI:** 10.3390/jcm14145103

**Published:** 2025-07-18

**Authors:** Marie-Louise Marschalek, Rodrig Marculescu, Christian Schneeberger, Julian Marschalek, Marlene Hager, Robert Krysiak, Johannes Ott

**Affiliations:** 1 Department of Obstetrics and Gynecology, Clinical Division of Gynecologic Endocrinology and Reproductive Medicine of the Medical University Vienna, 1090 Vienna, Austria; marie-louise.marschalek@meduniwien.ac.at (M.-L.M.); christian.schneeberger@meduniwien.ac.at (C.S.); julian.marschalek@meduniwien.ac.at (J.M.); marlene.hager@meduniwien.ac.at (M.H.); 2 Department of Laboratory Medicine, Institute of Immunology, Medical University Vienna, 1090 Vienna, Austria; rodrig.marculescu@meduniwien.ac.at; 3 Department of Internal Medicine and Clinical Pharmacology, Medical University of Silesia, Medyków 18, 40-752 Katowice, Poland; rkrysiak@sum.edu.pl

**Keywords:** polycystic ovary syndrome, mental health, coping, psychiatric disorders, depression, anxiety

## Abstract

**Background and Objectives**: Polycystic ovary syndrome (PCOS) is associated with an elevated risk of impaired mental health and psychiatric disorders, such as depression and anxiety. Physical factors like weight and hirsutism, as well as psychological factors, such as self-esteem and coping strategies, are all known to have an influence on mental health status. **Aim:** To assess psychological symptoms in women with and without PCOS, by use of the well-established, validated self-report questionnaire: Symptom Checklist-90-Revised (SCL-90); to determine the reliability of the SCL-90 for assessment of PCOS patients. **Design**: Prospective case-control study. **Methods**: Psychological symptoms were assessed using the German version of the SCL-90 in 31 PCOS women and 31 healthy controls. To test the impact of various parameters on numerical outcome parameters, correlation analyses were conducted. **Results**: PCOS women revealed significantly increased SCL-90 scores in seven out of the nine subscales (hostility subscale, anxiety subscale, depression subscale, paranoid ideation subscale, psychoticism subscale, somatization subscale, interpersonal sensitivity subscale, obsessive compulsive subscale), as well as in all three global indices (*p* < 0.05). SCL-90 scores were significantly positively correlated with perceived total stress and perceived helplessness and significantly negatively correlated with perceived self-efficacy (*p* < 0.05). **Conclusions**: PCOS women experienced higher levels of psychological symptoms including depressive and anxiety symptoms. Higher perceived stress, higher perceived helplessness and lower self-efficacy were associated with more psychological symptoms. Hence, there is a need to support PCOS women with their emotional regulation and coping strategies.

## 1. Introduction

Polycystic ovary syndrome (PCOS) is a common female endocrine disorder, with a prevalence of 12% among women of reproductive age, affecting millions of women worldwide [1]. PCOS is considered a combined metabolic-endocrine disorder and is primarily characterized by many things including insulin resistance, low-grade inflammation, and hyperandrogenism. However, the symptoms go beyond physical health, as women with PCOS are at higher risk of developing psychological stress due to dissatisfaction with their physical appearance, resulting in a perceived negative body image, and consequently impaired quality of life. These factors have an impact on mental health, and psychiatric disorders such as depression and anxiety may occur. These latter psychiatric disorders are known to be more commonly encountered in PCOS women [2,3] imposing a substantial health care burden. Over the past years numerous systematic reviews and meta-analyses have found that the quality of life in women with PCOS is reduced and that anxiety and depression are more regularly observed amongst women affected by this condition [4]. Similarly, Li et al. revealed that adolescents with PCOS also experienced more severe depressive symptoms than those without PCOS [5]. In particular, the inability to conceive leading to fertility stress in PCOS women is associated with depression [6,7].

In the general population, any chronic illness is a stressful condition and may result in the advent of depression and anxiety. Mental health in women with PCOS is, on the one hand, affected by obesity [8], and on the other hand, affected by the androgen related symptoms, namely hirsutism, menstrual irregularities, acne, and subfertility. Hirsutism is known to be significantly correlated with mental health status [9]. These relevant physical aspects, including weight and hirsutism, as well as psychological factors, such as self-esteem, and sociocultural pressures, all have an influence on body image. The various factors determine the extent of stress, which is significant as depression and anxiety are acknowledged as being linked to higher stress levels [10].

As a result, in order to recognize the priorities of the needs of patients, the 2023 updated “International evidence-based guideline for the assessment and management of polycystic ovary syndrome PCOS” highlights the need to take quality of life into account as a factor in PCOS research, as well as the application of correct methods in clinical care [11]. The updated guideline further recommends the inclusion of psychological evaluation, and the routine screening of all women for mental health issues at the time when PCOS is diagnosed.

Although the prevalence of, and severity of, depressive and anxiety symptoms in PCOS women has increased, international surveys have reported that most women consider that psychological issues continue to be under-recognized when PCOS is diagnosed, and sadly less than 5% of women are satisfied with the level of counseling and emotional support [12].

The Symptom Checklist-90-Revised (SCL-90) is a well-established, validated self-report questionnaire for measuring a range of psychological and psychiatric disturbances [13]. It involves nine scales, which entail Somatization, Obsessive-Compulsive, Interpersonal Sensitivity, Depression, Anxiety, Hostility, Phobic anxiety, Paranoid Ideation and Psychoticism. The SCL-90 is a universally recognized method of assessment of psychological distress in clinical practice and research. The instrument aims to contribute to accurately assessing one’s subjective experience of psychopathology, without requiring the involvement of mental health professionals [14]. In PCOS women, the SCL-90 has been used in some studies [15,16,17], however, depressive and anxiety symptoms in PCOS women have so far been mainly assessed by different means in the literature such as the Beck Depression Inventory or the Hospital Anxiety and Depression Scale (HADS) [18]. Despite being a validated tool, the SCL-90 has not commonly been used for assessment of women with PCOS. Hence, the reliability of SCL-90 in PCOS women could be questioned.

As a result of the frequent occurrence of mental health disorders in women with PCOS, and the lack of standardized screening tools, our aim was to evaluate psychological symptoms in normal and overweight women with PCOS women compared to healthy women without PCOS by a validated questionnaire. Furthermore, our aim was to determine the reliability of SCL-90 in PCOS patients by correlating SCL-90 results with the standard tool of quality life, the Short form-36 (SF-36) [19].

## 2. Materials and Methods

### 2.1. Study Design

We conducted this prospective case-control trial at the Clinical Department of Gynecologic Endocrinology and Reproductive Medicine of the Medical University of Vienna, Austria. It received the approval of the ethics committee of the Medical University of Vienna (IRB number 1804/2016). Written informed consent to participate was obtained from all participants.

### 2.2. Patient Selection

Non-infertile women diagnosed with PCOS with an age ranging between 18–40 years. were screened for eligibility. PCOS diagnosis was confirmed according to the revised Rotterdam criteria [20], by the presence of two of the following three criteria: (1) biochemical/clinical signs of hyperandrogenism; (2) polycystic ovaries on transvaginal ultrasonography; (3) ovulatory dysfunction such as oligomenorrhea or amenorrhea [20]. Exclusion criteria were non-classic congenital adrenal hyperplasia and a PCOS therapy in the previous three months prior to recruitment. Participants were recruited at the Department of Gynecologic Endocrinology and Reproductive Medicine of the Medical University of Vienna. All women had a standard medical assessment including laboratory testing. Further, participants completed the self-administered questionnaires.

Control subjects volunteered to participate and were healthy women with a regular cycle, no indication of clinical/biochemical hyperandrogenism, without the use of any hormonal contraception. None of the controls had any known medical condition.

### 2.3. Measures

Psychological disturbances were assessed with the German version [21] of the SCL-90, an established screening instrument including 90 items with a five-point scale (0 = not at all, 4 = extremely) with nine scales (Somatization, Obsessive-Compulsive, Interpersonal Sensitivity, Depression, Anxiety, Aggression, Phobia, Paranoid Ideation and Psychoticism) and three global categories (Global Severity Index GSI, Positive Symptom Distress Index PSDI, Positive Symptom Total PST). GSI reflects the overall distress, PSDI the intensity of distress and PST the total number of stress-inducing symptoms. The scores were calculated according to the guidelines [21]. Raw scores were calculated by dividing the sum of scores for a scale by number of items in the scale. Global Severity Index was computed by summing the scores of all nine scales, then dividing by the total number of responses. Positive Symptom Total counts the total number of questions rated above 1 point. The Positive Symptom Distress Index was calculated by dividing the sum of all item values by the PST. Higher SCL-90 scores indicate higher morbidity. Although the SCL-90 is not specifically validated, other studies have used the SCL-90 questionnaire in PCOS patients [15,16,17,22].

The German version of the 10-item Perceived Stress Scale (PSS-10) was used to evaluate perceived stress. This questionnaire measures the degree by which life events are appraised as stressful, uncontainable or overwhelming. [23]. Answers are scored on a 5-point scale (0 = never to 4 = very often). The PSS is linked to less life satisfaction comprising two factors: perceived helplessness and perceived self-efficacy. The PSS-10 score is the sum of all perceived helplessness items after having deducted the score of the perceived self-efficacy items.

The German version of the Short Form-36 (SF-36) was used to assess quality of life. The questionnaire is an established and validated 36-item tool with 8 subdomains: “Physical functioning”, “Physical role functioning”, “Emotional role functioning”, “Energy fatigue”, “Emotional wellbeing”, “Social functioning”, “Pain” and “General health” [19]. The scores were converted to a 0–100 scale, according to the guidelines. A higher score indicates higher quality of life and improved health status.

We evaluated the clinical parameters relevant to PCOS. Body mass index (BMI) was calculated by weight/height squared (kg/m^2^). Hirsutism was detected by use of the Ferriman-Gallwey method [24]. The global acne grading system was used to assess acne severity [25]. To detect polycystic ovarian morphology (PCOM) > 12 follicles per ovary on ultrasound were needed. Ultrasound examinations were performed via “Aloka Prosound 6” ultrasonography machine and “UST-9124 Intra Cavity transducer” (frequency range 3.0–7.5 MHz; Wiener Neudorf, Austria) [26].

All patients had a hormonal investigation on a blood sample during the early follicular phase visit (cycle days 2–5 which included total and free testosterone [ng/mL], dehydroepiandrosterone sulfate (DHEAS) [µg/mL], androstenedione [ng/mL], sex hormone binding globulin (SHBG) [nmol/L], luteinizing hormone (LH)/follicle-stimulating hormone (FSH) ratio, prolactin [ng/mL] and anti-Mullerian hormone (AMH) [ng/mL]. The biochemical analyses were performed at the Department of Laboratory Medicine, Medical University of Vienna, in accordance with ISO 15189 quality standards [27]. Prolactin, DHEAS and cortisol were measured by electrochemiluminescence immunoassays (ECLIA) using Cobas e 801 immunologyanalyzers (Roche Diagnostics, Mannheim, Germany) [26]. Free testosterone was calculated using the free androgen index [FAI = 100 × (total testosterone/SHBG]. Homeostasis Model Assessment (HOMA) for insulin resistance (IR) was calculated with the formula (fasting plasma glucose [mg/dL] × fasting insulin [mU/L])/405 with a cut-off of >2.5 [28].

### 2.4. Statistical Analysis

These findings are based on a secondary analysis within our previously published investigation on saliva stress markers in PCOS women [29]. The sample size was established in this first investigation within our study.

Median and interquartile range are used to present numerical parameters, whereas categorical parameters are presented as numbers and frequency. Differences between groups were evaluated by the unpaired *t*-test (in case of a normal distribution) or the Wilcoxon rank sum test for numerical parameters Relationships between numerical parameters were calculated using the Pearson correlation. Statistical analyses were performed in SPSS 24.0 (IBM, Vienna, Austria). Binary logistic regression models were used to compare questionnaire results between PCOS patients and groups, when results were matched for age and BMI. A *p*-value < 0.05 was considered statistically significant.

## 3. Results

Thirty-one women with PCOS and 31 control subjects were included in the study. Basic clinical characteristics and results of hormonal testing in PCOS women and healthy controls are presented in Table 1. The PCOS group revealed a higher BMI, a higher Ferriman Gallwey score, higher testosterone, DHEA-S, LH, prolactin and AMH levels, an increased FAI, and higher rates of PCOM on ultrasound (*p* < 0.05).

PCOS women revealed significantly increased SCL-90 scores in seven out of the nine subscales (hostility subscale, anxiety subscale, depression subscale, paranoid ideation subscale, psychoticism subscale, somatization subscale, interpersonal sensitivity subscale, obsessive compulsive subscale), as well as of all three global indices (GSI, PST, PDSI) (Table 2). Furthermore, women with PCOS revealed significantly lower quality of life in all SF-36 modules apart from “Pain”, as reported previously [29].

Correlation analyses were performed between the SCL-90 domains and PCOS specific parameters (Table 3). No significant correlations were found between SCL-90 scores and PCOS specific parameters.

Correlation analyses were performed between the SCL-90 domains and perceived stress (Table 4). Apart from the anxiety scale all SCL-90 scales were significantly positively correlated with perceived total stress and perceived helplessness and significantly negatively correlated with perceived self-efficacy (Table 4).

Correlation analyses were performed between the SCL-90 domains and SF-36 domains (Table 5). All SCL-90 domains showed overall convergence and negative correlations with most of SF-36 domains.

## 4. Discussion

In our study, PCOS patients exhibited all typical PCOS-related features. About 52% of the PCOS women were overweight/obese with a median age of 25 years. PCOS patients revealed increased testosterone, DHEA-S and LH levels (Table 1). However, the PCOS group were significantly younger and had a higher BMI compared to the control group. Thus, taking this into account, all results of the questionnaires were corrected for differences in age and BMI (Table 2).

SCL-90 subscales negatively correlated with SF-36 domains, relating to lower quality of life in women with PCOS. This indicates the reliability and validity of the SCL-90 in PCOS patients.

Women with PCOS had significantly higher scores on most of the SCL-90 subscales and on all global indices in comparison to the control group. These analyses were corrected for age and BMI. This conforms with the findings of other studies, such as Guo et al. who reported elevated depression and anxiety scores of SCL-90 in PCOS patients before investigating the effect of pioglitazone and metformin on psychological distress [30]. Similarly, Hahn et al. demonstrated significantly higher SCL-90 scores in PCOS patients compared with the German norm, prior to treatment with metformin [31]. Our results underscore the general agreed consensus that women with PCOS have a higher propensity towards emotional stress and psychological disturbances [2,4,32]. Interestingly none of the PCOS specific parameters were correlated with SCL-90 scores.

Notably, SCL-90 scores were positively correlated with perceived total stress and perceived helplessness and significantly negatively correlated with perceived self-efficacy. This finding is plausible, as more helplessness leads to more psychological symptoms, likewise less self-efficacy may cause more psychological symptoms.

Generally, in order to be able to live with a disease, patients use a variety of coping strategies to manage the stress resulting from the condition. These strategies are a set of individual cognitive and behavioral efforts which aim to change or to adapt to a stressful situation and thereby help to reduce distress [33]. Coping strategies can be divided into two types of problem solving, they may be active, aiming to eliminate and alter the source of stress, or avoidant, aiming to reduce aversive emotions and thereby regulating the emotional consequences. Active strategies are considered to be superior leading to a better ability to adapt to living with the disease [34]. Individual coping strategies influence the level of stress experienced by the patient and the lack of an ability to adapt to stressful life situations has a major impact on the quality of life. Previous research demonstrates that PCOS women show deficits in coping with stress and experience difficulties with coping with their condition [35,36,37]. In particular, PCOS women use passive and adaptive strategies to cope with stress, which are known to be a predictor of high levels of anxiety and depression [38]. Anxiety levels in PCOS women are closely related to the level of coping skills [39]. Dysfunctional coping may lead to not only higher perceived stress levels, but, more seriously, to psychological symptoms such as depression, paranoid ideation, somatization and obsessive compulsive disorder.

To the best of our knowledge, this study is one of few studies investigating the validated SCL-90 questionnaire in PCOS women. Further strengths of our study are the excellent participant retention. However, several limitations have to be addressed. The control group had a lower BMI than the PCOS participants. We realize that the homogenous population may affect and limit the generalizability of our trial. The small sample size needs to be mentioned. Further, the sample size calculation was determined based on the different outcome obtained in our first investigation. It is also possible that unknown medical conditions might have existed in the control group, which could have an impact on the main outcome. Furthermore, one could argue that neither the SCL-90 nor the SF-36 are validated for PCOS women. However, the SCL-90 has been used before in PCOS patients and in many other fields for assessing mental health in different disorders and non-psychiatric populations without being specifically validated for this purpose [40,41,42].

## 5. Conclusions

This study demonstrates that women with PCOS experience significantly higher levels of psychological symptoms including depression, anxiety, and elevated perceived stress, compared to women without PCOS. Moreover, our findings reveal that greater perceived stress and feelings of helplessness, along with lower self-efficacy, are strongly linked to increased psychological symptomatology. In addition, the SCL-90 results correlated with the standard SF-36 outcomes in this PCOS population. These results underscore the need for intervention programs focused on emotional regulation and stress management and highlights the importance of routine mental health screening in PCOS populations. Future research should not only further assess and manage perceived stress and psychological symptoms in this group but also validate the SCL-90 against other diagnostic tools. Such efforts will refine our understanding and treatment of psychological distress in women with PCOS, paving the way for more effective clinical strategies.

## Figures and Tables

**Table 1:** Clinical and biochemical characteristics in PCOS women and controls.

	PCOS Patients	Controls	*p*
Age (years)	25 (22;30)	28 (24;31)	0.074
BMI (kg/m^2^)	24.9 (21.5;35.5)	21.5 (19.5;23.9)	0.022
Insulin resistance (HOMA-IR > 2.5)	20 (64.5)	-	-
Ferriman Gallwey Score	10 (5;15)	0 (0;0)	<0.001
GAGS	4 (4;11)	0 (0;6)	0.133
LH (mlU/mL)	12.6 (8.5;14.6)	5.2 (3.4;7.1)	<0.001
FSH (mlU/mL)	5.6 (4.9;7.5)	5.9 (4.7;8.1)	0.899
LH: FSH ratio	2.18 (1.51;3.09)	0.93 (0.55;1.4)	<0.001
Testosterone (ng/mL)	0.51 (0.39;0.72)	0.25 (0.16;0.31)	<0.001
SHBG (nmol/L)	35.3 (29.1;73.7)	82.3 (68;114.6)	0.001
Free androgen index	1.15 (0.72;2.53)	0.29 (0.20;0.38)	<0.001
DHEA-S (µg/mL)	3.17 (2.53;4.33)	2.19 (1.81;2.80)	<0.001
Prolactin (ng/mL)	13.1 (8.9;17.8)	9.5 (8.3;13.1)	0.026
AMH (ng/mL)	7.83 (5.94;11.00)	3.01 (2.03–4.07)	<0.001
Presence of polycystic ovarian morphology on ultrasound	25 (80.6)	4 (12.9)	<0.001

Data are presented as median (IQR) for numerical parameters and as numbers (frequencies) for categorical parameters. Abbreviations used: BMI, body mass index; HOMA-IR, homeostasis model assessment index of insulin resistance; GAGS, global acne grading system; LH, luteinizing hormone; FSH, follicle stimulating hormone; SHBG, sexual hormone binding globulin; DHEA-S, dehydroepiandrosterone-sulfate; AMH, anti-Mullerian hormone.

**Table 2:** Results of the SCL-90 questionnaire in PCOS women and controls.

	PCOS Patients	Controls	OR (95% CI)	*p*	*p* *
Hostility	0.33 (0.17;1.00)	0.33 (0.17;0.50)	0.3620 (0.727;18.032)	0.361	0.116
Anxiety	0.50 (0.20;0.80)	0.20 (0.10;0.50)	12.810 (1.501;109.346)	0.010	0.020
Depression	0.73 (0.38;1.31)	0.38 (0.15;0.69)	9.374 (1.827;48.087)	0.003	0.007
Paranoid ideation	0.50 (0.17;0.88)	0.17 (0;0.33)	9.291 (1.796;48.068)	0.001	0.008
Phobic anxiety	0.14 (0;0.18)	0 (0;0.14)	1.418 (0.090;22.277)	0.726	0.804
Psychoticism	0.30 (0.10;0.73)	0 (0;0.20)	523.188 (15.416;17,756.094)	<0.001	<0.001
Somatization	0.63 (0.42;1.10)	0.25 (0.17;0.67)	6.262 (1.440;27.227)	0.003	0.014
Interpersonal sensitivity	0.44 (0.22;1.14)	0.44 (0.11;0.78)	3.713 (1.156;11.926)	0.097	0.028
Obsessive compulsive	0.70 (0.50;1.13)	0.40 (0.30;0.80)	5.605 (1.254;25.048)	0.005	0.024
GSI	0.61 (0.35;0.83)	0.32 (0.17;0.51)	97.523 (5.406;1759.218)	<0.001	0.002
PSDI	1.53 (1.25;1.95)	1.30 (1.16;1.50)	9.958 (1.591;62.342)	0.014	0.014
PST	34.5 (22.3;45.8)	21.0 (11.0;34.0)	1.085 (1.031;1.142)	0.002	0.002

Data are presented as median (IQR). * *p* was adjusted for age and BMI. Abbreviations used: GSI, global severity index; PSDI, positive symptom distress index; PST, positive symptom total.

**Table 3:** Correlation analyses: PCOS-specific parameters versus SCL-90 domains in PCOS women only.

	Anxiety		Depression		Paranoid Ideation		Somatization		Obsessive Compulsive		GSI	
	r	*p*	r	*p*	r	*p*	r	*p*	r	*p*	r	*p*
Age (years)	0.189	0.308	−0.118	0.535	0.253	0.178	0.176	0.351	−0.198	0.295	0.079	0.677
BMI (kg/m^2^)	0.029	0.879	−0.155	0.413	0.013	0.944	0.087	0.647	−0.034	0.860	−0.157	0.407
Ferriman Gallwey Score	0.001	0.998	−0.198	0.293	−0.003	0.989	0.062	0.744	0.025	0.895	−0.207	0.273
Free androgen index	−0.148	0.428	−0.359	0.052	−0.073	0.702	−0.012	0.948	−0.224	0.235	−0.328	0.077
AMH (ng/mL)	0.244	0.185	0.248	0.187	−0.038	0.844	0.152	0.422	0.188	0.321	0.289	0.121

All correlations were tested using the Spearman rank analysis. Abbreviations used: BMI, body mass index; AMH, anti-Mullerian hormone; GSI, global severity index.

**Table 4:** Correlation analyses: Perceived stress scale versus SCL-90 domains in PCOS women only.

	Anxiety		Depression		Paranoid Ideation		Somatization		Obsessive Compulsive		GSI	
	r	*p*	r	*p*	r	*p*	r	*p*	r	*p*	r	*p*
PSS 1	0.040	0.763	0.331	0.010	0.336	0.009	0.163	0.214	0.280	0.031	0.339	0.008
PSS 2	−0.005	0.967	0.386	0.002	0.461	<0.001	0.261	0.042	0.277	0.031	0.446	<0.001
PSS 3	0.055	0.672	0.290	0.023	0.200	0.122	0.319	0.012	0.266	0.038	0.361	0.004
PSS 4	0.100	0.441	−0.222	0.085	−0.169	0.193	−0.189	0.144	−0.181	0.163	−0.236	0.068
PSS 5	0.043	0.741	−0.355	0.005	−0.291	0.023	−0.123	0.346	−0.236	0.076	−0.341	0.007
PSS 6	0.196	0.130	0.475	<0.001	0.348	0.006	0.229	0.075	0.431	0.001	0.551	<0.001
PSS 7	0.115	0.379	−0.168	0.196	−0.148	0.254	−0.280	0.029	−0.160	0.219	−0.243	0.059
PSS 8	0.070	0.590	−0.454	<0.001	−0.340	0.007	−0.344	0.007	−0.366	0.004	−0.476	<0.001
PSS 9	0.178	0.169	0.247	0.055	0.338	0.008	0.132	0.312	0.133	0.308	0.305	0.017
Total PSS = PSS 10	0.126	0.333	0.428	<0.001	0.448	<0.001	0.424	0.001	0.375	0.003	0.509	<0.001
Perceived helplessness subscale	0.114	0.382	0.483	<0.001	0.503	<0.001	0.348	0.006	0.403	0.001	0.565	<0.001
Perceived self-efficacy subscale	0.110	0.398	−0.395	0.002	−0.103	0.428	−0.292	0.022	−0.302	0.018	−0.423	0.001

All correlations were tested using the Spearman rank analysis; Abbreviations used: PSS, Perceived Stress Scale, GSI, global severity index.

**Table 5:** Correlation analyses: SF-36 domains versus SCL-90 domains in PCOS women only.

	Interpersonal		Depression		Anxiety		Hostility		Phobic Anxiety		Paranoid Ideation		Psychoticism		GSI		PSDI	
	r	*p*	r	*p*	r	*p*	r	*p*	r	*p*	r	*p*	r	*p*	r	*p*	r	*p*
Physical functioning	−0.132	0.478	−0.190	0.306	−0.405	0.024	−0.171	0.358	−0.241	0.191	−0.280	0.126	−0.462	0.009	−0.424	0.020	−0.348	0.059
Role functioning	−0.089	0.634	−0.151	0.416	−0.205	0.268	−0.040	0.831	−0.207	0.264	−0.011	0.954	−0.259	0.160	−0.397	0.030	−0.245	0.192
Role emotional	−0.425	0.017	−0.472	0.007	−0.460	0.009	0.418	0.019	−0.338	0.063	−0.307	0.093	−0.621	<0.001	−0.565	0.001	−0.481	0.007
Energy fatigue	−0.370	0.040	−0.434	0.015	−0.399	0.026	−0.255	0.166	−0.399	0.026	−0.341	0.061	−0.349	0.054	−0.458	0.001	−0.484	0.007
Emotional wellbeing	−0.637	<0.001	−0.745	<0.001	−0.656	<0.001	−0.457	0.010	−0.272	0.138	−0.534	0.002	−0.441	0.013	−0.697	<0.001	−0.697	<0.001
Social functioning	−0.275	0.135	−0.117	0.532	0.072	0.700	0.053	0.776	0.017	0.929	−0.218	0.238	−0.118	0.527	−0.154	0.417	−0.311	0.095
Pain	−0.277	0.132	−0.420	0.019	−0.427	0.017	−0.604	<0.001	−0.322	0.078	−0.258	0.161	−0.382	0.034	−0.475	0.008	−0.432	0.017
General health	−0.171	0.357	−0.325	0.074	−0.447	0.012	−0.364	0.044	−0.201	0.278	−0.339	0.062	−0.377	0.036	−0.399	0.029	−0.258	0.168

All correlations were tested using the Spearman rank analysis; Abbreviations used: GSI, global severity index. PSDI, positive symptom distress index.

## Data Availability

Anonymized data will be shared on request from any qualified investigator.

## Abbreviations

The following abbreviations are used in this manuscript:

SCL-90	Symptom Checklist 90
PCOS	Polycystic ovary syndrome
HADS	Hospital Anxiety and Depression Scale
IRB	Institutional Review Board
GSI	Global Severity Index
PSDI	Positive Symptom Distress Index
PST	Positive Symptom Total
PSS	Perceived Stress Scale
FAI	Free androgen index
DHEA-S	Dehydroepiandrosterone sulfate
LH	Luteinizing hormone
FSH	Follicle-stimulating hormone
AMH	Anti-Mullerian hormone
SHBG	Sex hormone binding globulin
HOMA	Homeostasis model assessment
IR	Insulin resistance
BMI	Body mass index
ECLIA	Electrochemiluminescence immunoassays
GAGS	Global acne grading system
PCOM	Polycystic ovarian morphology
IQ	Interquartile range
